# Strategic resilience in an adaptive SERE & H.E.A.T. course – an explorative study of human performance and resilience

**DOI:** 10.3389/fpsyt.2026.1777679

**Published:** 2026-04-10

**Authors:** Sabrina Ziehr, Philipp Hans Merkt

**Affiliations:** 1 Department of Disaster Prevention and Crisis Management (bzgk.de), Research and Education Center for Extraordinary Tactical Situations and Strategic Resilience (18_RECESS), University of Applied Sciences gem. Trägergesellschaft, Idstein, Germany; 2 Faculty of Health (Department of Human Medicine), University Witten Herdecke gGMBH, Witten, Germany

**Keywords:** 18E – Überleben Land, explorative study, SERE & H.E.A.T., human performance, strategic resilience, peer group

## Abstract

**Objectives:**

The current study aims to evaluate the fitting accuracy of the Model of Strategic Resilience (Merkt and Ziehr) in relation to a specific SERE and H.E.A.T. scenario in Germany using the course “Überleben Land – HEAT (18E)” as a case study.

**Methods:**

Psychosocial, cognitive, and physical data of six participants were collected. These were distance, pace, and heart rate, tracked by chest straps and multifunctional watches. To evaluate psychosocial and cognitive data, semi-structured qualitative interviews were conducted, supplemented with a short questionnaire during the escape phase. The evaluation was carried out by triangulating the collected data.

**Results:**

The participants describe challenges in their human performance concerning all three categories of the Model of Strategic Resilience. In addition, resources are mentioned, especially for the psycho-emotional and cognitive sectors, that allow the participants to accomplish the scenario. Particularly, they highlight the harmonious atmosphere in the group, the rational assessment of the situation, and the usefulness of acquired knowledge and skills.

**Conclusion:**

The current study shows that the Model of Strategic Resilience is a useful tool to detect physical, cognitive, and psychological parameters of human performance, and to identify resources and strategies that address resilience in different courses, such as H.E.A.T., SERE, or the German Commando Course.

## Introduction

**Figure 1:**
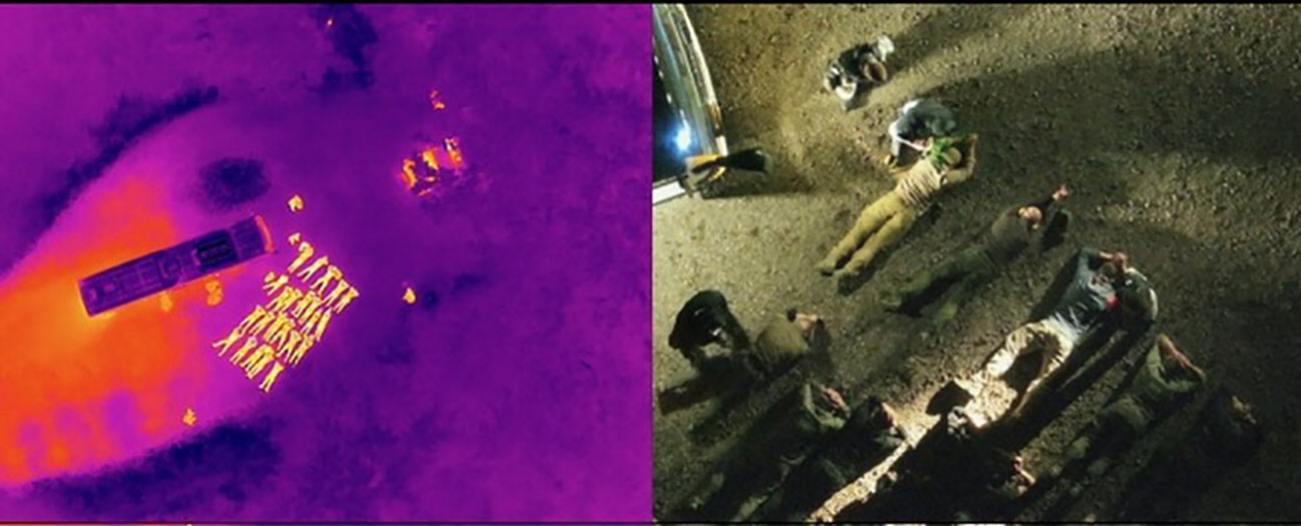
Überleben Land – HEAT (18E). Section (C) hijacking of the course participants and the test persons by trained police personnel. Right: Thematic technique of a drone image in a stress posture. Left: Search and closing of the sensory organ. (Source: HSF-BZGK, 2025).

Hybrid threats, such as terrorism, are steadily increasing (1) and pose challenges for those involved in such operations. Personnel are required to perform at a high level even under adverse conditions. One model that attempts to map human performance and the resilience of individuals in the context of special operational situations is the Strategic Resilience Model (2). It claims to be able to map resilience in a wide variety of contexts based on the components of physicality, cognition, and psyche. An important field of research in human performance and strategic resilience is the study of working in high-risk areas. One possible scenario is a hostage-taking situation followed by isolation/detention and rescue by personnel recovery teams (**Figure 1**). This is trained within a continuing education course in accordance with H.E.A.T, SERE, and German Commando Course standards, with students and external participants at the Department of Disaster Prevention & Crisis Management (BZGK), Idstein, Germany.

### Strategic resilience

**Figure 2:**
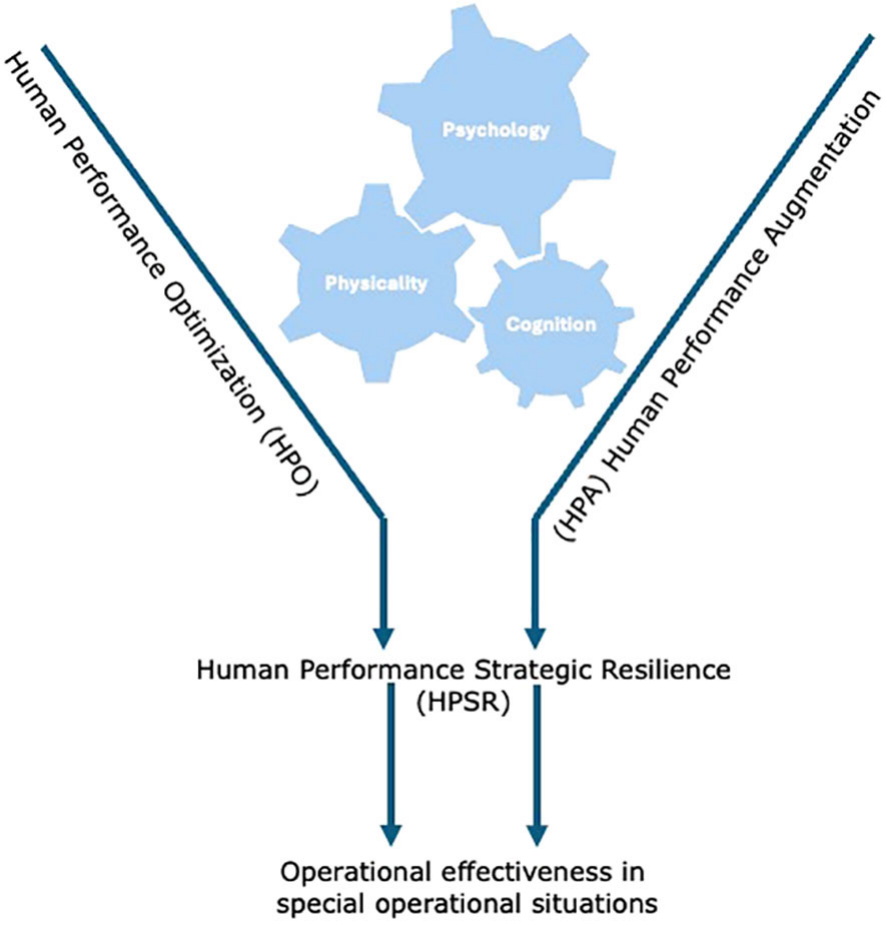
Model of strategic resilience in human performance by Merkt and Ziehr (2).

The Model of Strategic Resilience by Merkt and Ziehr (2) aims to cover relevant markers of resilience in three domains: physical, cognitive, and psychological/psychosocial factors (**Figure 2**). The physical domain initially includes general physical condition in terms of fitness. It also describes body functions that interact while coping with stress, for example, the functioning of the metabolic system and sleep (3, 4). Furthermore, vital parameters such as blood pressure or heart rate can be used to describe stress, despite alpha-amylase or cortisol being the traditional markers of stress (5). Cognitive function includes the functioning of the sensory organs, such as hearing, smelling, sensing, and tasting, measured by the MARC-Test (6). In addition, the general performance in attentiveness and concentration is examined. Overall, cognitive functioning is important for decision-making decisions (7). Being cognitively alert in special operational situations is a key factor for successful performance (8).

The psychological or psychosocial sector deals with emotional coping with situations and the feelings individuals are exposed to. Relevant factors are hope, a sense of involvement, and having a support network (3). Beyond that, there is a strong connection between psychological and cognitive elements, because aspects such as self-efficacy, self-esteem, or attitude to life cannot be clearly assigned to one domain alone. This domain reflects the personal mindset, which depends on personal attitudes, feelings, and emotions, and has a significant impact on how a person experiences a special situation (9). The importance of mindset in special situations is reflected in statements by the German Armed Forces, which expect defiance from soldiers deployed in a state of defence at Germany’s eastern border (10).

The Model of Strategic Resilience by Merkt and Ziehr (2) assumes that all three domains must interact in the best positive way to have a positive impact on the human performance of the actors in high-risk tasks. Following this, an optimized outcome of the special operational situation is determined. At the Department of Danger Prevention and Crisis Management at Fresenius University of Applied Science, the model is used as a framework for conceptualizing study programmes such as the Tactical Emergency Medicine for Disaster Management and Counterterrorism (11).

### Human performance

**Figure 3:**
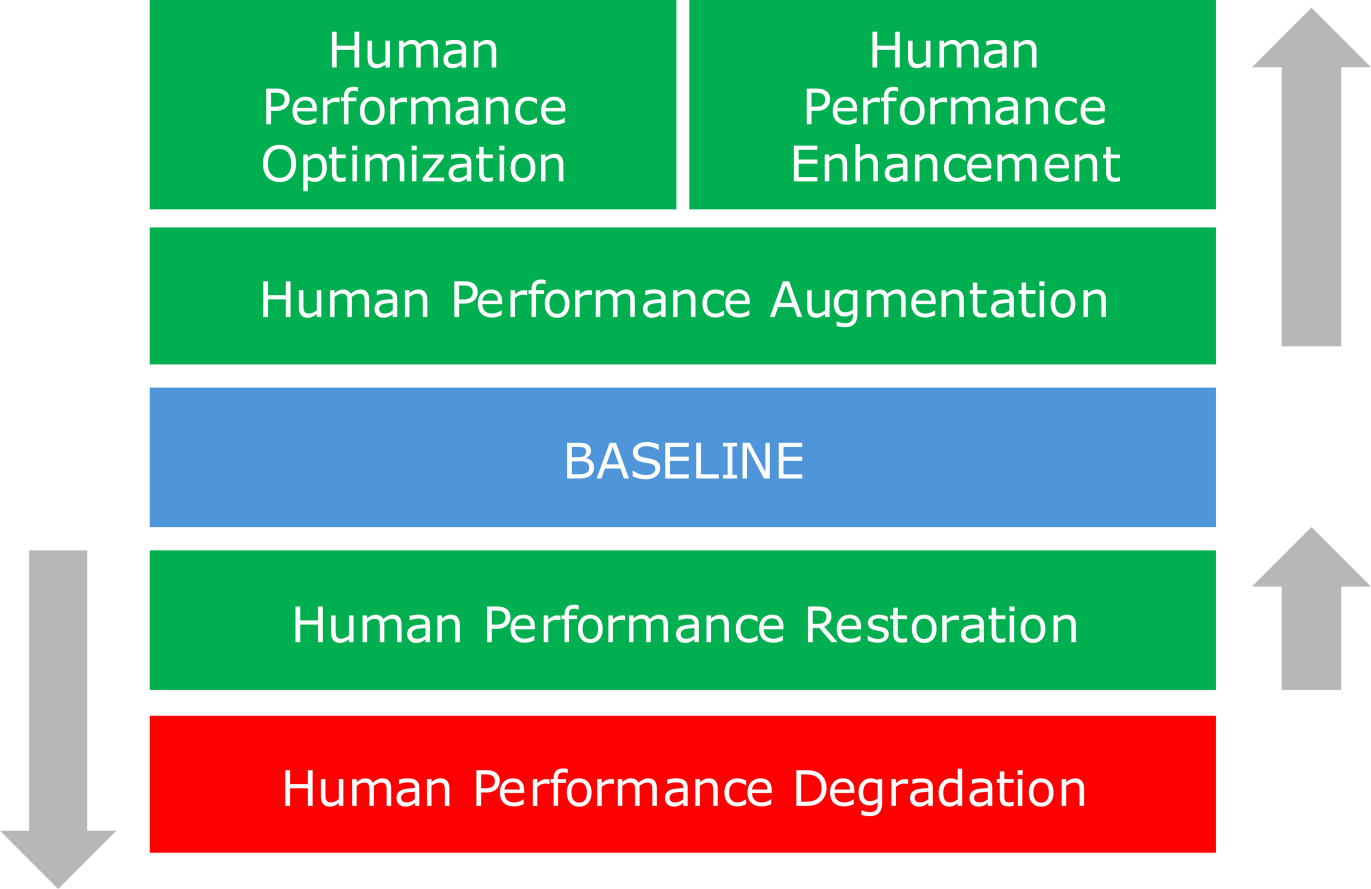
Human performance and its subtypes (in accordance with 15).

Across society, including governments, athletes, and the general service sector, there is a continuous drive for improvement, greater efficiency, profit, and prestige. Therefore, all sectors strive for optimization. In the framework of strategic resilience, focus is placed on individual human performance. In that context, the term Human Performance describes an individual’s ability to successfully complete a specified task in a manner that meets mission demands (12). There are several influencing factors on human performance, for example, sleep (4) or probiotics (13). Psychological aspects also influence human performance and mindset (14). Furthermore, the cognitive load is studied as an important indicator of a soldier’s ability to operate effectively (8). An international project, the multinational capability development campaign, has described different subtypes of human performance. These include human performance modification, restoration, augmentation, optimization, enhancement, and degradation (15). Human performance modification (HPM) refers to any change in human performance, whether deterioration or improvement. Degradation and restoration deal with performance below the personal basic level. Human performance augmentation (HPA) describes an expansion of the person’s human performance. It is subdivided into human performance optimization (HPO) and human performance enhancement (HPE). While optimization refers to improving existing skills and performance, enhancement is characterized by technological features that exceed natural human performance (15). **Figure 3** provides an overview of the different subtypes.

Merging all aspects of human performance is a complex construct of being capable of taking action, and it is obvious that human performance and resilience are closely interconnected. Strengthening resilience has an impact on human performance, and vice versa.

The model of operational effectiveness in special operational situations includes training formats such as:

- H.E.A.T. (Hostile Environment Awareness Training) prepares civilians, such as those part of NGOs, and military personnel for deployment in high-risk areas, focusing on safety, behavior during abductions, and first aid.
- SERE-C (Survival, Evasion, Resistance, Escape) trains individuals to survive in hostile territory, avoid capture, resist interrogation, and escape captivity.
- The German Commando Course (EKL 1) is an intensive military course designed to build physical and mental resilience, navigation skills, and combat readiness under extreme conditions.

All three course concepts aim to increase operational success in special operational situations. In doing so, physical performance, cognition, and psychological resilience are challenged in different dimensions of operational environments (16). The learning strategies of these courses differ according to the mission profile. EKL 1, with a duration of approximately four weeks, differs significantly in intensity and content from SERE Bravo and SERE Charlie (17), and from the H.E.A.T. course (18).

In its core mission profile, EKL 1 focuses on operating as a unit behind enemy lines and going back to the own platoon. This includes techniques of ambush, evasion, infiltration, and engagement (19). In contrast, SERE (Bravo & Charlie), with a total duration of approximately two to three weeks, focuses on resistance in hostage situations and escape in hostile territory. Survival techniques are only taught in two-person teams, not in groups (17). The core competency lies in orientation towards a reception procedure within a (dynamic) rescue zone.

By contrast, the H.E.A.T. course, with a duration of four to six days, focuses on awareness and the instruction of fundamental survival skills. For the previously mentioned course concepts, prior military basic training is required, which represents a different entry level regarding physical performance, cognition, and psychological resilience compared to H.E.A.T. Regarding access requirements from a human performance perspective, there are also significant differences concerning medical entry examinations and the assessment of basic physical fitness, including performance ergometry (20), such as the occupational medical examination according to G26.3 for heavy respiratory protection (fire service).

**Figure 4:**
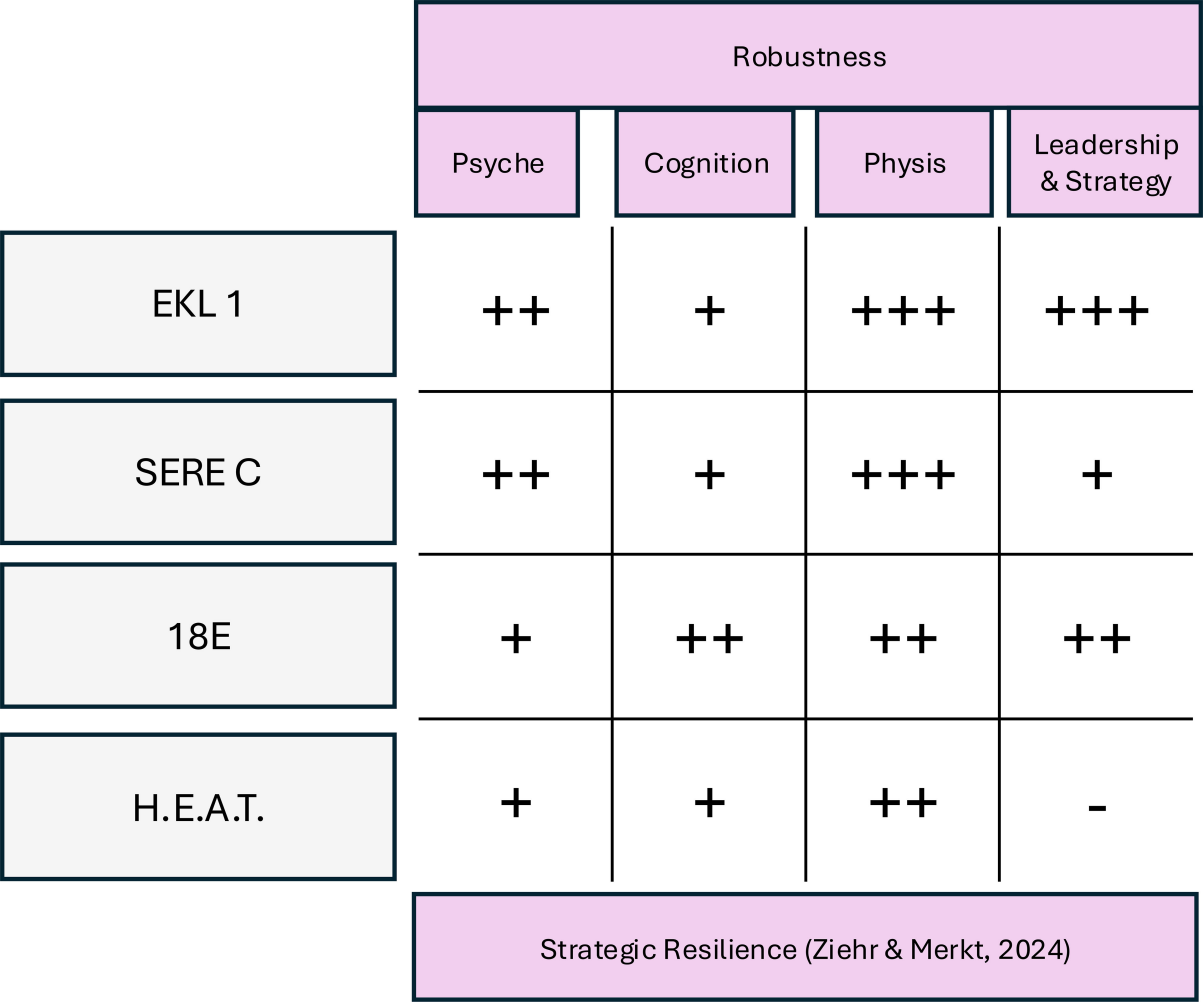
Comparative overview of the degree of robustness in the dimensions of psyche, cognition, physique, and leadership & strategy in selected course formats.

Within the framework of a modular continuing education program across two master’s degree programs:

- Crisis and Emergency Management (KNM) and,
- Tactical Emergency Medicine for Disaster Management and Counterterrorism (Master Medic/Master Physician) (TENuK),

the BZGK developed a continuing education concept in 2019 based on EKL 1, SERE, and H.E.A.T. The continuing education program “Überleben Land – HEAT (18E)” is oriented towards the quality criteria of the Star of Bravery and thus emphasizes stress-resistant operational capability under extreme environmental conditions (21). Leadership under stress is taught as part of strategic resilience, with psychological and cognitive influencing factors playing a central role in group leadership (2). The development of self-leadership and peer leadership under stress follows both military psychological foundations (22) and a proportionate adaptation to motivational needs according to Maslow (23) or, similarly, to the Full Range Leadership Model (24). **Figure 4** provides a comparative overview of the four courses described above. They are assessed by rating the level of stress (+++ to −) relating to the four dimensions psyche, cognition, physique and leadership & strategy.

The aim of the study is to investigate whether the 18E-Course fits in the Model of Strategic Resilience (2) and which of its components are likely to be necessary for completing the course.

## Methods

Depending on the presented theoretical background and the updated state of knowledge, the current study aims to evaluate the fitting accuracy of the Model of Strategic Resilience in Human Performance by Merkt and Ziehr (2), because to date no empirical data are available to determine whether the model is suitable for practical application. As a potentially stressful training that addresses all three domains of the model, the specific training 18E in Germany is used for empirical investigation. The 18E combines several aspects of the existing courses EKL1, SERE C, and H.E.A.T., allowing different stress-inducing events to be integrated into a single course. It highlights hostage situations, evasion, and leadership under stress, incorporating different navigation skills and survival elements within a short time frame. 

To pursue this aim, an exploratory study with a small sample size and a focus on qualitative research methods (semi-structured interviews) supplemented by quantitative measurements (physical parameters) was chosen. This approach meets the requirements of qualitative data triangulation by using different data collection methods within the same research context, enabling an in-depth and descriptive analysis of the subject (25, 26).

### Participants

Participants were recruited via convenience sampling. They were either students of the courses at the Department of Danger Prevention and Crisis Management at Fresenius University of Applied Sciences or external participants who wanted to complete the 18E course. Inclusion criteria were defined as:

Students or external participants enrolled in the 18E course.
Positive medical check (internal and functional orthopedic check).
Physical performance equivalent to the occupational medical examination according to G26.3 for heavy respiratory protection (fire service).
Professional or voluntary engagement in danger prevention or disaster events.
Legal adult age.
Voluntary participation in the study.
Written informed consent.

All subjects confirmed voluntary participation through written informed consent before starting the course.

The participants were six individuals enrolled in the 18E course in summer 2025. All participants were male and aged between 20 and 50 years. All participants were experienced in training courses such as the 18E course. All of them took had participated in a preparatory course at the Department of Danger Prevention and Crisis Management at Fresenius University of Applied Sciences approximately half a year before. All participants successfully completed the course during the summer.

### Material

Demographic data such as age, sex, and experience were collected using a short questionnaire. The qualitative data were collected through semi-structured guided interviews. The interview was divided in seven themes: open start, overall recapitulation, hostage, isolation, extraction and flight, sleeping, and final comments. Each theme began with an open question to encourage a maximum of open and detailed responses. In addition, follow-up questions (“how did you feel about it” or “can you explain a little more”) and clarification prompts were included to gain a deeper insight into the participant’s perceptions (27, 28). For data reliability, all interviews were audio recorded using a tablet device.

Quantitative data were collected using smartwatches and chest straps, based on their successful application in a pretest in the same setting in 2023 and in comparable study settings at the BZGK, such as research on stress in air rescue operations. According to the manufacturer, the multisport smartwatch Garmin fēnix 6 Pro enables continuous second-by-second wrist-based heart rate monitoring, in addition to recording of respiratory rate, oxygen saturation (SpO_2_), and movement data, with a battery life of up to 36 hours in GPS mode. To increase measurement sensitivity and validity, the watch was supplemented with the Garmin HRM-Pro chest strap, which provides ECG model-based heart rate measurement with high temporal resolution and delivers more precise signals, particularly under physical load. The parallel acquisition of heart rate data via optical wrist-based sensors and ECG measurement via the chest strap allows mutual plausibility checking of the measurement data and reduces motion-related artefacts, while distance covered is reliably determined using multi-GNSS tracking (GPS, GLONASS, and Galileo) (29).

### Data collection

The short questionnaire was completed prior to the course. The qualitative interviews were conducted during the course after the extraction phase near the participants’ resting places in the woods. All interviews were conducted by the same researcher, who adapted the interview guide to the chronology of each respondents’ narrative and used further prompts where necessary. The duration of the interviews ranged between 19 and 49 minutes. None of the interviews were interrupted.

The measurement devices used were multisport watches of the Garmin fēnix 6 Pro type, in addition to the supporting chest strap Garmin HRM-Pro. The devices were worn continuously throughout the entire measurement period and operated in continuous training mode without measurement interruptions. The measurement time points were:

- Resting measurement (without intervention).
- Theoretical instruction (last will and testament).
- Hijacking.
- Hostage-taking (conduct after capture).
- (I.) Evasion (Escape).
- (II.) Evasion (Survival).
- Recovery by own forces.

To minimize measurement errors, all participants and the professional group companions were informed about the use of the watches. Furthermore, the technical functioning was checked by the assistant researcher whenever possible.

**Table 1** provides an overview of the measurement phases using theGarmin fēnix 6 Pro, including the operational context, terrain characteristics, environmental conditions, threat levels, and intervention domains across the training and operational sequence.

**Table 1:** Physical measurements – methods.

Measurement	A	B	C	D	E	F	G
Content	Resting measurement (without intervention)	Theoretical instruction (last will and testament)	Hijacking	Hostage-taking (conduct after capture)	(I.) Evasion (escape)	(II.) Evasion (survivial)	Recovery by own forces
Day (approx.)	1 I (2000–2300)		1–2 I (2400–2000)		2–3 (2000–2100)		3 (2100–0100)
Time (min/h)	120/2	120/2	150/2.5	1.200/20	360/6	1.800/30	120/2
Terrain profile	Urban (Classroom)	Urban (Classroom)	Country road	Deserted building	movement along a stream through dense forest // gross route: 7.8 Km	assessment of difficult terrain with partial elevation sections // gross route: 19.8 Km	open areas with embankments
Environment	Indoor (Room temperature)		Outdoor (12C°), Bus (28C°)	Cold Room (16C°)	Outdoor (20–24C°)	Outdoor (20–29C°)	Outdoor (18–20C°)
Target accuracy	non		hight level of enemy activity	middle level of enemy activity	high level of enemy activity	middle level of enemy activity	low level of enemy activity
Intervention	non	Psyche / Cognition	Psyche / Cognition	Psyche / Cognition / Physis	Psyche / Cognition / Physis /Leadership & Strategy	Psyche / Cognition / Physis /Leadership & Strategy	Leadership & Strategy

The devices were checked in the field within fixed, predefined time windows and charged using power banks, with all measurement data stored centrally on the watch and fully downloaded after completion of the course.

### Data interpretation

Demographic data were analyzed using Microsoft Excel.

The qualitative interviews were transcribed using Microsoft Word and MAXQDA. All personal data that could identify the interview partners were pseudonymized. Original and edited material were stored separately, with no access granted to third parties. Interpretation was conducted by the first author using qualitative content analysis (30). The software MAXQDA 24 (31) was used for organizing the qualitative data analysis. Initially, themes derived from the interview guide and the Model of Strategic Resilience (2) were fixed as codes (deductive coding). After the first round of coding, these codes were differentiated into subcodes based on the participants’ statements (inductive coding). For example: The interviewer asked (deductive code): “What are you experiencing regarding your cognition?” The interviewee’s answer, “Staying focused after sleep deprivation so that you don’t make any mistakes, or at least not as many or as serious ones”, was coded as cognitive functioning (inductive code). Some codes were merged, while others were deleted. All codes were described with coding rules and anchor examples. Ultimately, the whole code system comprised of six codes as headlines with 29 subcodes. The six headlines were: comments on the course, coping, group, psyche, cognition, and physique. **Table 2** shows the complete code system.

**Table 2:** Complete code system for the interpretation of the interviews.

Theme/code (deductive)	Subcode (inductive)
Physis	overall well-being
	resource
	circumstances
	motion
	nature’s call
	sleep
	nutrition
	weather conditions
Cognition	self-determination
	expectation
	experience
	mindset
	rational viewpoint
	sensory organs
	cognitive functioning
	stress
Psyche	evaluation of state
	positive feelings
	negative feelings
	uncertainty
	sense of safety
	feeling of freedom
Coping	strategies
	tools
Group	constellation
	potential for conflict
	personal position
	leadership
	cooperation & collaboration
Comments on the course	

Overall, 258 text passages were marked and categorized.

The datasets, with an average recording duration of 65 h/3.600 min (gross), were extracted from the Garmin watch using the manufacturer’s proprietary software provided by Garmin GmbH and transferred into an Excel-based database. The data were prepared in a participant-specific structured format and subjected to statistical analysis. In addition, a data triangulation of.

- total distance,
- total time (measurement),
- maximum heart rate,
- average heart rate,
- steps,
- time in motion

was conducted following the six-participant approach, to systematically interlink physiological, spatial, and temporal measurement dimensions and to ensure interpretative robustness.

## Results

The results of the study are presented in relation to the Model of Strategic Resilience with its three dimensions, namely, physical, psychosocial, and cognitive conditions. In addition, coping strategies and group structure are revealed. Quantitative data are included in the physical dimension. The analysis concludes with the superior themes of coping strategies and group structure.

### Physical dimension

The physical parameters, namely, total distance (km), maximum heart rate (bpm), average heart rate (bpm), steps (approx.), and time in motion (min), were recorded for all six participants over a period of three days, with a total net measurement duration of 65 hours. **Table 3** lists all parameters.

**Table 3:** Physical measurement – results.

Measurement		A	B	C	D	E	F	G
Content		Resting measurement (without intervention)	Theoretical instruction (last will and testament)	Hijacking	Hostage-taking (conduct after capture)	(I.) Evasion (escape)	(II.) Evasion (survivial)	Recovery by own forces
Day (approx.)		1 I (2000–2300)		1–2 I (2400–2000)		2–3 (2000–2100)		3 (2100–0100)
Time (min/h)		120/2	120/2	150/2,5	1.200/20	360/6	1.800/30	120/2
Terrain profile		Urban (Classroom)	Urban (Classroom)	Country road	Deserted building	movement along a stream through dense forest//Gross route: 7,8 Km	assessment of difficult terrain with partial elevation sections//Gross route: 19,8 Km	open areas with embankments
Environment		Indoor (Room temperature)		Outdoor (12C°), Bus (28C°)	Cold Room (16C°)	Outdoor (20–24C°),	Outdoor (20–29C°)	Outdoor (18–20C°)
Target accuracy		non		high level of enemy activity	middle level of enemy activity	high level of enemy activity	middle level of enemy activity	low level of enemy activity
Intervention		non	Psyche/Cognition	Psyche/Cognition	Psyche/Cognition/Physis	Psyche/Cognition/Physis/Leadership & Strategy	Psyche/Cognition/Physis/Leadership & Strategy	Leadership & Strategy
Person	Measurement	A	B	C	D	E	F	G
1	total distance (km)	<0,1	<0,1	1.2	1.7	15.4	28.3	4.3
	maximum heart rate (bpm)	75	82	124	136	153	130	112
	average heart rate (bpm)	63	65	74	68	115	108	98
	steps (aprox)	<150	<150	bus drives	2,070	20,950	41,890	6,320
	time in motion (min)	<15	<15	25	65	290	540	105
2	total distance (km)	<0,1	<0,1	1	0.5	15.2	29.1	4.1
	maximum heart rate (bpm)	67	75	90	75	135	165	105
	average heart rate (bpm)	59	63	71	68	90	83	79
	steps (aprox)	<150	<150	bus drives	1,035	20,540	42,250	6,305
	time in motion (min)	<15	<15	19	33	289	555	98
3	total distance (km)	<0,1	<0,1	1.1	1.1	16.1	28.3	4.3
	maximum heart rate (bpm)	91	85	110	104	167	137	113
	average heart rate (bpm)	68	68	73	67	104	98	87
	steps (aprox)	<150	<150	bus drives	1,887	21,202	41,889	6,310
	time in motion (min)	<15	<15	21	55	315	536	100
4	total distance (km)	<0,1	<0,1	1.2	2.3	15.1	29	4.1
	maximum heart rate (bpm)	86	82	124	131	172	168	97
	average heart rate (bpm)	74	70	79	72	91	83	77
	steps (aprox)	<150	<150	bus drives	3,089	20,950	42,280	6,325
	time in motion (min)	<15	<15	22	98	286	540	105
5	total distance (km)	<0,1	<0,1	1.2	0.9	15.3	28.3	4.3
	maximum heart rate (bpm)	78	67	89	83	165	134	108
	average heart rate (bpm)	71	63	69	67	88	91	90
	steps (aprox)	<150	<150	bus drives	986	21,030	41,798	6,101
	time in motion (min)	<15	<15	25	40	287	545	100
6	total distance (km)	<0,1	<0,1	0.9	2.1	15.4	28.3	4.4
	maximum heart rate (bpm)	72	81	97	110	144	171	107
	average heart rate (bpm)	69	69	73	71	96	101	98
	steps (aprox)	<150	<150	bus drives	3,324	21,206	41,890	6,320
	time in motion (min)	<15	<15	18	105	290	540	101

The measurements are divided into sections A to G. Section A represents the input measurement at rest. Sections A and B took place in the lecture hall (No. 1 – 63 bpm; No. 2 – 59 bpm; No. 3 – 68 bpm; No. 4 – 74 bpm; No. 5 – 71 bpm; No. 6 – 69 bpm). In these sections, all participants consistently show low stress, as indicated by average heart rate (bpm).

Sections C and D were carried out outdoors and in non-heated buildings. Here you can see the work done by all six test subjects. Under stress in section D (deserted building), the number of steps is significantly increased compared to the section B (urban classroom). All six participants were found to exert themselves beyond baseline levels, which is reflected in increased time in motion corresponding to the number of steps.

In section E, the measurement was carried out under significant physical stress and a high level of simulated enemy activity, using the maximum heart rate (No. 1 – 153 bpm; No. 2 – 135 bpm; No. 3 – 167 bpm; No. 4 – 172 bpm; No. 5 – 165 bpm; No. 6 – 144 bpm). The simulated escape phase was completed with luggage (20–25 kg) under tactically guided movement in a stream (approximately 2 km) and in rough terrain over approximately 8 km. The breakthrough and orientation phase from one hiding place to another in Section F, which occurred mainly at night in cooler temperatures, showed a balanced physical performance overall. However, two participants reached maximum heart rates (No. 2 – 165 bpm; No. 4 – 168 bpm), above the average of the other four participants.

The planned march in section E (movement along a stream through dense forest, gross route: 7.8 km) deviated considerably from the total distance (km) actually covered by all six participants. For example, distances reached 15.4 km for participant No. 1 and 15.1 km for participant No. 4. A comparable phenomenon was observed in section F (assessment of difficult terrain with partial elevation sections, overall route: 19.8 km), in which a significantly higher total distance (km) was also measured in all six participants, reaching up to 29 km.

Section G, the admission procedure by one’s own forces, showed no notable deviations special features. It was shown that the participants’ values returned to their initial values in the absence of physical stress and a low level of enemy activity.

In addition to physical measurement by the watches and chest straps the subjective perspective was explored through qualitative interviews. It can be separated into one general and six specific themes. In general, the participants reported some distress in their overall well-being. For example, they felt confused about their physical condition due to being less active, less fit, and somewhat clumsy or in pain. Being physically active by choice was described as a valuable resource to improve well-being during the course, especially during isolation and shortly after it. Participants reported: “Well, it was kind of liberating to walk” (No. 1) or “Lately, when we knew what our mission was, that we now have to walk 1,8 km through that river, our smile was back and we felt motivated” (No. 2). Restrictions were reported in sleep, nutrition, and toilet access. Time for sleep and the comfort of having food readily available were lacking. Toilet use was limited during the isolation phase because participants had to ask to be escorted to the toilet. Participant 6 explained it as a challenge: “Not to go to the toilet by own choice, when I wanted to”. Additionally, the weather had an impact on the participants. Expected rain or low temperature was described as further challenges. Participant 5 said: “I can be with every weather, but if anything is damp or moist. [sic] I felt so jolly glad that there was no rain last night”.

### Cognitive dimension

The emerging themes can be divided into two sectors: mindset and cognitive functioning. Cognitive function was affected by losing senses or having reduced sensory capabilities during capture, for example, seeing and hearing. Participants felt challenged by being masked with glasses or wearing headsets. Participant 2 pointed out: “So, I have to say, if ears and eyes are offline, I am fast running on empty” [sic], which led to uncertainty. Participant 4 also reported feeling burdened. Others, such as Participant 6, experienced an increased reliance on other sensory perceptions: “I realized that there are other things functioning. Not that I have a much better olfactory sense but one focus on other helpful things”. Cognitive function was mentioned by only a few respondents. Participant 6 reported the feeling worse decision-making and having trouble concentrating due to sleep deprivation. This compares to the statements relating to personal mindset, which was identified by the majority of participants as a resource for overcoming challenges during the course. Participants with experience in high-risk professions,(e.g., police or military) referred to existing coping mechanisms, such as anticipating what would happen next. “Nonetheless, it is a bridge to know how the procedure normally is going” [sic] (No. 3). Others referred to their personal environment by mentally going home to their families (No. 2), reminding themselves that it was only a training that couldbe stopped at any time, or focusing on the end of the course (No. 3, 4, 5, 6). Furthermore, the participants described it as helpful to have a positive mental attitude and the ability to evaluate situations rationally in challenging situations. Participant 1 explained: “I think the point is not to give up but to have strategies and tactics in mind how to handle upcoming situations in a way not to feel helpless or powerless”. Participant 3 said: “The mental attitude, the mindset to say: I want to do this, and I have to do this”. However, this is not implicit, as Participant 1 stated: “Exhausting. Yes. That the mindset rests free to decide ‘Okay, let’s do it, it is funny’”. It goes along with the general anticipations participants have when facing the course. Being well prepared for the situation and having plans and ideas on how to handle it was described as an important resource (No. 3, 4). In contrast to positive expectations, some of the participants reported fear or uncertainty, which can compromise personal performance although one knows that it can be handled. Participant 5 described: “The interesting point is that the fear mostly is bigger than the reality. What about fear, one has worse in mind, but being in the situation, it is running” [sic]. Besides all the presented positive effects, stress was also experienced. For example, it was reflected in feeling of loss of control (No. 1), dissatisfaction with personal performance (No. 5), and the feeling of being in a doom loop, doing the same things again and again (No. 5).

### Psychosocial dimension

The psychosocial dimension is predominantly depicted by emotions and feelings. Although some participants did not report any negative effects (No. 1) and felt well, happy, and satisfied (No. 2) during the interview, others reported discomfort during the course. They described *negative feelings* such as being bored (No. 2, 4) or fed up (No. 5) because of missing activities. Additionally, not being called by one’s own name, due to being numbered as a fictive hostage, had a negative impact. Feeling unsafe due to uncertainty of the following tasks, feeling helpless, or being hungry were also reported as negative (No. 4). *Positive feelings* were associated with being active, such as crossing a river (No. 1), getting out of isolation (No. 4), fulfilling difficult tasks (No. 2), or experiencing the shining sun (No. 5). The group constellation was also positive, as Participant 1 explained: “I breathed a sigh of relief and thought: Thank God, that is a good combination”. Being with oneself (No. 1) or recognizing familiar faces (No. 4) during the course gave a sense of security.

### Coping strategies

The participants reported several helpful strategies to deal with the different situations during the course:

- Spirituality, such as religion (No. 1) or meditation (No. 2, 4).
- Thinking of positive experiences, such as family (No. 1) or daily life at home (No. 3).
- A personal positive mindset (No. 3).
- Being experienced, e.g., hiking (No. 2) or having overall life experience (No. 2, 4).
- Support from the group (No 4).

Participant 6 found it helpful to wear headphones, because it provided the opportunity to be as with by himself.

### Group structure

Inductive coding identified five subheadings belonging to the group structure. Although the constellation of the group was not important before (No. 1, 3), it had an impact during the course. Being in a good team made it easier to undergo the challenges (No. 1, 4, 5). Potential for conflict was the heterogeneous constellation of personnel from the military, police, and civil society (No. 1, 2, 4). In connection with that, leadership and communication were experienced as potentially difficult. Because of different professional or cultural imprints, the way of leading and communicating was varied. Some participants could be better guided than others (No. 4). In addition, the different characters (No. 6) and the outer circumstances (No. 4) could affect harmonious group settings. The personal position in the group was important and mostly described as being helpful for others (No. 1, 2, 3, 5, 6). Participant 3 described: “I want to support the group, help them succeed, fit in, and subordinate myself. Or sometimes take the lead. So, it depends on the situation we find ourselves in, and then we have to see how we can solve it together”. That goes along with Participant 5’s opinion: “Either I kick up a fuss and say ‘no, we are going to do it this way and that way’ or I say ‘yes, okay, we will do it the way you say and we will just see how it turns out’. That is what we did, and my colleague handled it really well, actually”. Participant 6 emphasized: “If I have to play the pack mule, then I am the pack mule”. Most statements pointed toward *cooperation and collaboration*. Although the participants had different experiences, the working progress was described as well-functioning. Helping each other, for example, with food and water or blankets against the cold, was crucial for the group’s success. Even though there were different characters, overall it was a team performance, because everyone looked out for one another (No. 2, 4, 5). “I think the greatest resource is still the ability to work in a team, to modularize these individual characters, but also to tie them together and connect them to the individual problems, and then to solve such a problem together” (No. 3).

## Discussion

The presented results are subject to some limitations. The main limitations are:

- Small sample size.
- Explorative design using qualitative research methods supplemented by physical measurements.

The sample contains six participants of the 18E course. They were all male. It is not possible to generalize the findings, but it provides an indication of whether continuing research can be considered useful. For small sample sizes and phenomena with limited empirical knowledge, qualitative research is an appropriate research method. Supporting qualitative data by objective measurements, such as examining physical parameters via chest strap and multisport watches, can strengthen the findings. By continuing the research, it could be beneficial to link qualitative and quantitative data more precisely. Despite the mentioned limitations of the study, it is possible to derive results that allow a first statement about the empirical basis of the model of strategic resilience (2).

**Figure 5:**
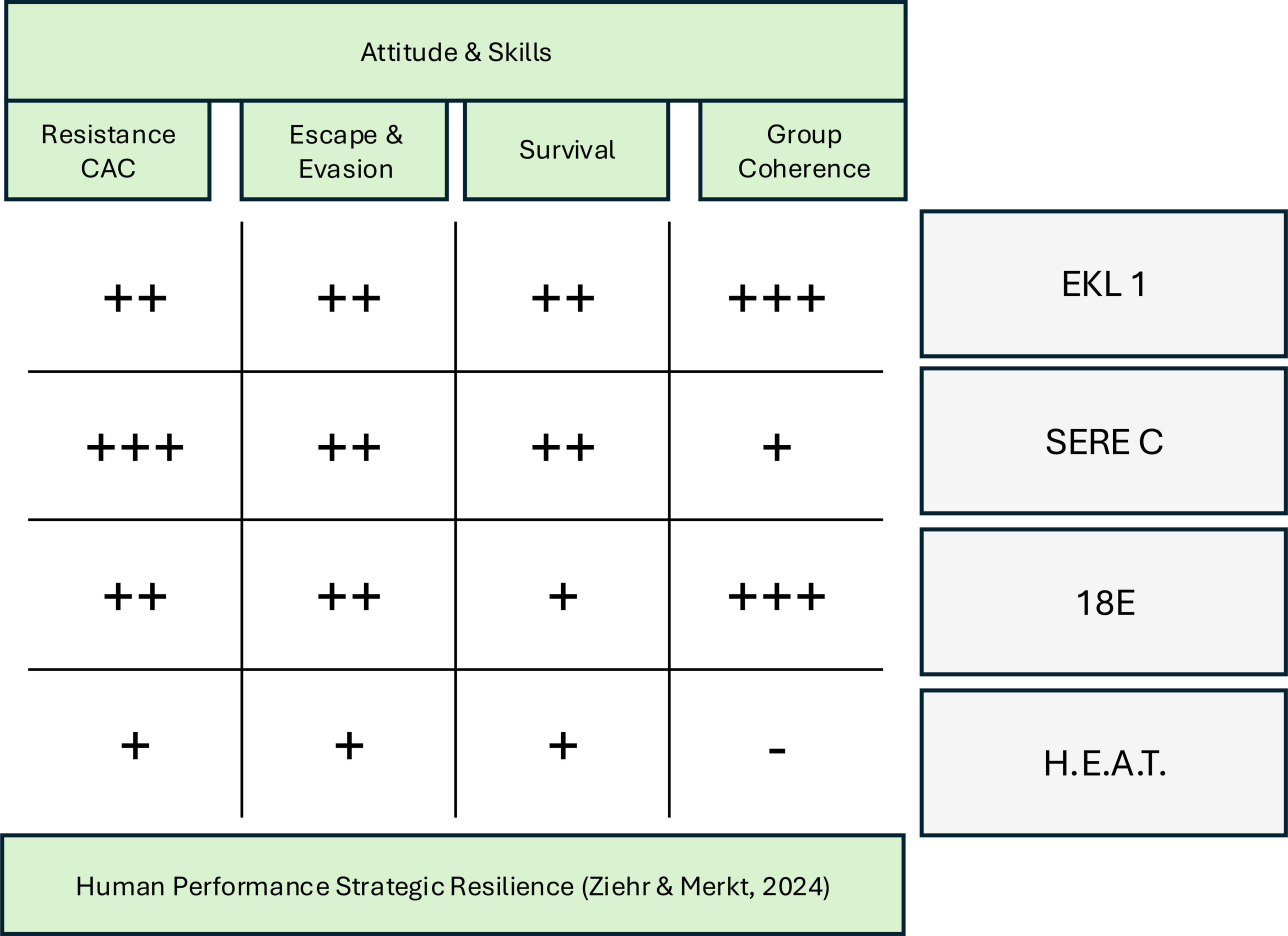
Comparative classification of selected training formats regarding human performance and strategic resilience.

Human Performance Optimization (HPO), together with Human Performance Enhancement (HPE) (15), describe the two axes of the capability profile for operational success in special situations. The aim of the methodological and didactic concept of the Überleben Land – HEAT (18E) course is to ensure preparation for special, potentially life-threatening operational situations, both individually and as part of a team, in line with the central course objectives of the German Commando Course, SERE Bravo/Charlie, and Hostile Environment Awareness Training. All four course formats mentioned above are self-contained in terms of content, and each is modelled according to the requirements of a specific hazardous situation. However, they all share the goal of enabling participants to reliably recall the necessary attitudes and skills in special situations under physical, cognitive, and psychological stress. **Figure 5** shows a comparative classification of selected training formats regarding the development of attitudes and skills in the areas of resistance (conduct after capture), escape and evasion, survival, and group cohesion under extreme stress. The qualitative assessment (+++ to −) illustrates the respective focus of the formats EKL 1, SERE C, Überleben Land – HEAT (18E) and H.E.A.T. in the context of Human Performance Strategic Resilience according to Ziehr and Merkt (2).

It should be emphasized that, compared to the other formats, the Survival Land – HEAT (18E) course represents the most balanced combination of these stress dimensions. It should also be noted that leadership and decision-making under stress in unfamiliar teams of armed and unarmed personnel take on particular significance. In addition, the course Überleben Land – HEAT (18E) does not work with classic termination criteria such as those applied in the German Commando Course or SERE Bravo/Charlie courses in the event of elimination due to professional misconduct. Instead, it guides participants through the mechanisms of the development field along the axes of HPO, specifically pushing them to the limits of strategic resilience and beyond. In this context, under stress in high-risk situations, the minimum individual performance within the team significantly determines the collective ability to act in terms of strategic resilience.

In accordance with the principle of learning in a practical spiral from the general to the specific (11), sensory experiences play a crucial role in perceiving, understanding, and coping with exceptional situations, and the neuro-mindset plays a central role in learning resistance (conduct after capture), escape and evasion, survival, and integration into an unknown or existing group. In this context, the performance and ability to act of the collective are largely determined by the weakest link, which means that individual deficits have a direct impact on the operational success of the entire team. At the micro level, the hippocampus should not be understood in isolation, but rather as the node or anchor point of a limbic-diencephalic neuron chain in the sense of the Papez circuit, consisting of, among other things, the hippocampus and fornix, corpora mammillaria, the anterior thalamic nuclei, and the cingulate and parahippocampal cortex areas, which contribute significantly to episodic memory formation and thus to learning from experience (32). In this context, learning strategic resilience promotes crucial learning patterns for action-relevant behavior in operational success. Emotionally and sensorily bound experiences support learning processes in addition to capacity-based and fragmented storage in the hippocampus and increase their situational availability (33). In addition, muscle memory and physical robustness are relevant selection factors. The exploratory study shows that basic fitness, the intrinsic urge to move, and environmental influences such as heat and cold are particularly important components in this context.

The presented study shows that the course 18E at the Department of Danger Prevention and Crisis Management at Fresenius University of Applied Sciences addresses each of the three dimensions in the Model of Strategic Resilience by Merkt and Ziehr (2). It offers the opportunity for self-experience in physical, cognitive, and psychosocial demands during participation. However, some of the participants do not dive deep into the scenario and do not take it as a chance for a borderline experience. They use the training setting as a form of mental and cognitive denial or escape. In contrast, the majority of the participants engage in the scenarios and deal with their own personal limits. They challenge their mindset, their physical state, and are open to engaging in group situations under pressure.

**Figure 6:**
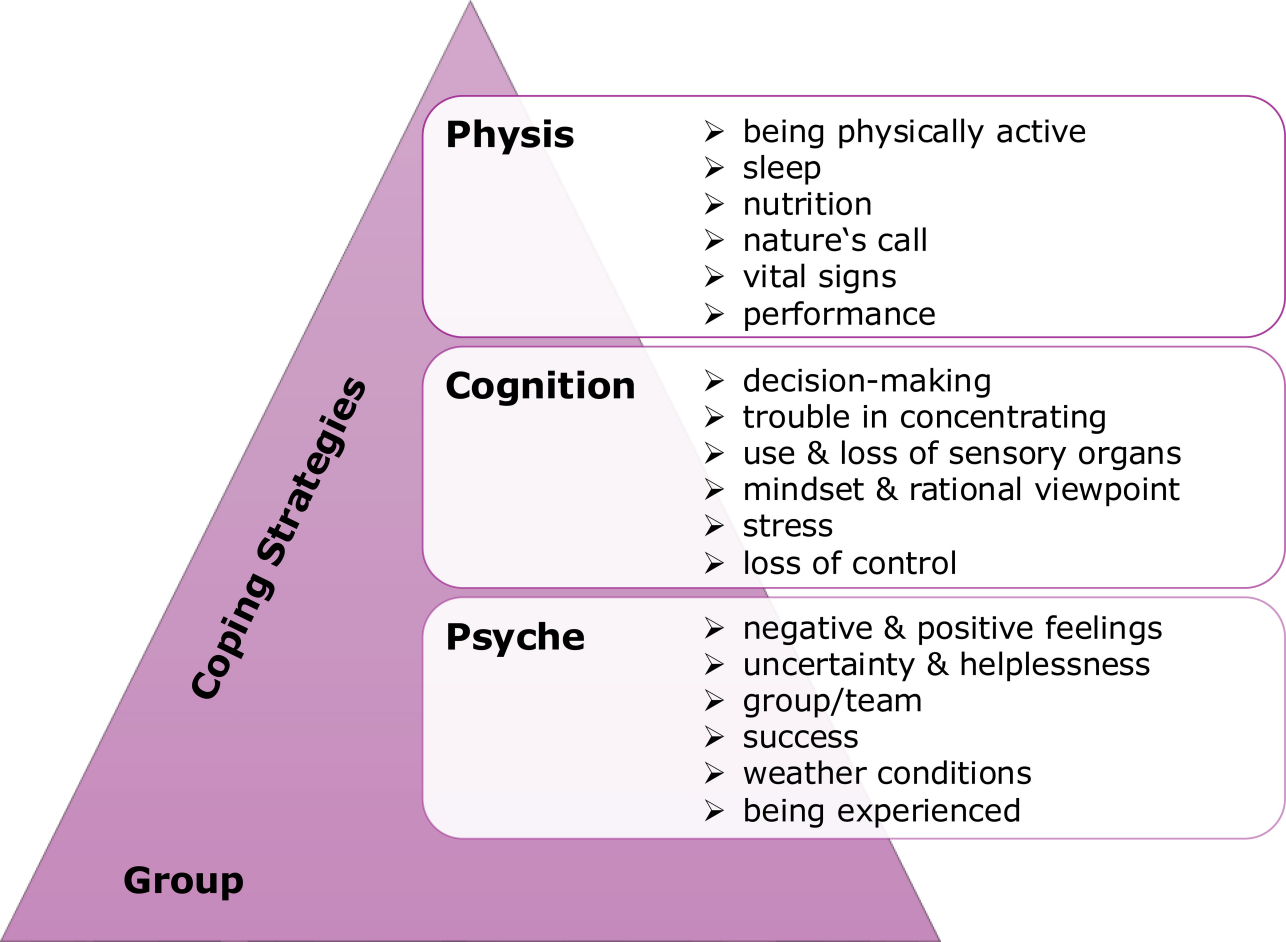
Strategic resilience in group settings.

For the cognitive dimension, participants reported challenges in decision-making due to sleep deprivation and partial loss of sensory input. It is consistent with the literature, which indicates that being less attentive or focused can have a negative impact on decision-making or task performance (6–8). Participants reported restrictions because of reduced sensory input, but also a concurrent improvement of the remaining senses. For research, it could be interesting to focus on it, as it may represent a potential entry point for specific training of human performance optimization. The personal mindset was mentioned as very important for completing several tasks during the course 18E. Staying positive, being self-reliant, and being conscious of one’s own strengths and limits are highly beneficial in overcoming the challenges. This strong effect can be observed in particular in section E, in which measurements under enormous physical stress and a high level of simulated enemy activity were reflected in the maximum heart rate (No. 1 – 153 bpm; No. 2 – 135 bpm; No. 3 – 167 bpm; No. 4 – 172 bpm; No. 5 – 165 bpm; No. 6 – 144 bpm). The capacitive physical and cognitive mindset of escaping together allowed for peak performance at the group level at this stage. In addition, from a motivational point of view, it should be noted that the group was only able to carry out orientation under limited low-light conditions, which further influenced the stress level (**Figure 6**), as can be seen in the results.

The mindset also links to the psychosocial dimension. During the 18E course, the participants experience various feelings ranging from very positive to negative. It is noteworthy that positive feelings and emotions are associated with physical activities such as movement. This can mainly be observed in the greater actual distances travelled (in km), depending on the assigned route sections. In their cognitive perception, the participants knew their actual marching performance, which differed from their original deviation in section F (assessment of difficult terrain with partial elevation sections, gross route: 19.8 km), in which all six participants also measured a significantly higher total distance (km) (No. 1 – 28.3 km; No. 4 – 29.0 km). That finding is consistent with research in human performance. For example, courses such as Total Force Fitness (34) focus on the interaction between physical, emotional, and cognitive states and improve human performance. However, a very important finding in the presented explorative study is the participants’ descriptions of coping strategies and the group setting. Both were explained as necessary tools for completing the course successfully. For that reason, the Model of Strategic Resilience by Merkt and Ziehr (2) should be expanded to include these two dimensions. **Figure 6** shows an adapted model in the context of group settings.

Besides personal fitness, described in the three dimensions of physis, cognition, and psyche, the necessary framework includes the ability to apply learned coping strategies and to develop a sense of coherence with the group, enabling individuals to overcome situations as a team, in which each member is responsible both for themselves for others.

All six participants reached the goal in section G, the admission procedure, by their own efforts, without failure, and as a cohesive group, whereby physical, cognitive, and psychological performance was maintained throughout. In the phases from Sections E to G, leadership was assumed by a team member on a rotating basis, who guided the remaining eight members through the individual sections. It is precisely under these conditions of leadership under stress in special situations that the group leader is particularly dependent on their strategic resilience. This shows that both experienced and inexperienced team members can make a significant contribution to the success of the mission in high-risk situations, regardless of their professional, cultural, or social background. The ability of the group leader to recognize these individual potentials, to promote them in a targeted manner, and to use them appropriately according to the situation thus proves to be a decisive factor for operational success.

These findings are consistent with current research in the field of Psychosocial Combat Readiness (PEF). In particular, the components of Functional Work Attitude and Comradeship and Team Orientation are represented in the presented explorative study. Although Mair and colleagues (35) could not draw a clear picture of its mechanisms, further research into these aspects appears to be worthwhile. The 18E course at the Department of Danger Prevention and Crisis Management at Fresenius University of Applied Sciences allows participants to work on their human performance by being physically, emotionally, and cognitively challenged. Further research to strengthen the presented findings and to determine the underlying causes, mechanisms, and consequences can be considered beneficial. It is clearly demonstrated that the 18E course fits within the Model of Strategic Resilience.

## Conclusion

The presented explorative study examines how the special 18E course at the Department of Danger Prevention and Crisis Management at Fresenius University of Applied Sciences fits within the Model of Strategic Resilience by Merkt and Ziehr (2). It highlights the opportunities offered to participants regarding their self-experience in human performance by completing the course. The course challenges participants in their physical, cognitive, and psychosocial condition. Therefore, it can be judged as a purposeful training for enhancing the strategic resilience of the participants and consequently their human performance. However, based on the presented data, it is not yet possible to conclusively determine whether human performance optimization and enhanced strategic resilience can be achieved through completion of the course. Due to the promising results, which indicate that each dimension of the Model of Strategic Resilience is addressed, the authors propose follow-up research to identify if and how an increase of human performance and strategic resilience can be measured. In addition, it seems useful to systematically research leadership behavior in terms of its effects on the group. Decisions to act are largely dependent on the performance of the respective team leader and have a direct influence on group dynamics, in particular in special situations.

## Data Availability

The raw data supporting the conclusions of this article will be made available by the authors, without undue reservation.
